# Knowledge, Attitude, and Practice of Pediculus Capitis Prevention and Control and Their Predictors among Schoolchildren in Woreta Town, Northwest Ethiopia, 2018: A School-Based Cross-Sectional Study

**DOI:** 10.1155/2020/3619494

**Published:** 2020-06-21

**Authors:** Henok Dagne, Awel Aba Biya, Amanuel Tirfie, Walelegn Worku Yallew, Zewudu Andualem, Baye Dagnew

**Affiliations:** ^1^Department of Environmental and Occupational Health and Safety, Institute of Public Health, College of Medicine and Health Sciences, University of Gondar (UoG), P.O. Box 196, Gondar, Ethiopia; ^2^Department of Human Physiology, School of Medicine, UoG, P.O. Box 196, Gondar, Ethiopia

## Abstract

**Background:**

*Pediculus capitis* is a human head lice infestation, a major public health issue that is most prevalent in resource-limited countries globally. The current study aimed to assess the knowledge, attitude, and practice of pediculus capitis prevention and control and their predictors among schoolchildren in North West Ethiopia.

**Methods:**

About 402 randomly selected schoolchildren from three schools in Woreta town participated in the study from April to June 2018. The outcomes of this study were knowledge, attitude, and self-reported practice of schoolchildren about *pediculus capitis* prevention and control. We used EPI Info 7.1 and SPSS 21 software for data entry and analysis, respectively. Binary logistic regression was employed to test the association of covariates with the outcome/response variables. Variables with a *p* value <0.2 during the bivariable binary logistic regression analysis were included in the multivariable binary logistic regression analysis. Variables with *p* value <0.05 were declared as significantly associated with outcomes.

**Results:**

The mean age of the study participants was 10.19 (±1.62) years. About 58.8%, 45.8%, and 78.6% of the schoolchildren had better self-reported *pediculus capitis* prevention knowledge, attitude, and practice, respectively. Age of children [9 to 11 years (AOR = 2.24, 95% C.I (1.10, 4.55)) and>12 years (AOR = 3.84, 95% C.I (1.56, 9.46))], better practice (AOR = 2.93, 95% C.I (1.39, 6.18)), and those who were not infested (AOR = 2.25, 95% C.I (1.14, 4.44)) were predictors of knowledge regarding *pediculus capitis* prevention. Better practice (AOR = 4.33, 95% C.I (1.69, 11.09)) and absence of infestation (AOR = 2.97, 95% C.I (1.64, 5.36)) were predictors of attitude of schoolchildren about *pediculus capitis* prevention. Number of students in a class [51 to 56 students per classroom, AOR = 4.61, 95% C.I (1.83, 11.67); 57 to 58 students per classroom, AOR = 8.18, 95% C.I (2.73, 24.46)], less than five family size (AOR = 2.37, 95% C.I (1.24, 4.54)), better knowledge (AOR = 2.93, 95% C.I (1.32, 6.50)), desirable attitude (AOR = 4.24, 95% C.I (1.60, 11.23)), and absence of infestation (AOR = 3.52, 95% C.I (1.22, 10.15)) were predictors of self-reported *pediculus capitis* prevention practice.

**Conclusion:**

The knowledge, attitude, and practice of schoolchildren regarding *pediculus capitis* prevention and control were not satisfactory. To bring change, intensive efforts on factors associated with the knowledge, attitude, and practice should be encouraged.

## 1. Background

*Pediculus capitis* is infestation by head lice that is a major public health problem globally which is most prevalent in low-income countries [1]. It is an omnipresent issue in children [2] in both schools and in the broader community [3]. *Pediculus capitis* can cause loss of sleep, irritation, pruritus, discomfort, secondary bacterial infections (such as impétigo and acute glomerulonephritis), and lymphadenopathy [4, 5]. The head lice are a potential vector for disease-causing organisms such as *Rickettsia prowazekii*, *Bartonella quintana*, and *Borrelia recurrentis* [6–11]. The methods to reduce the morbidity and prevalence of *pediculus capitis* include strategies targeted at increasing knowledge, changing attitudes and behaviors, and improving personal hygiene practice [6]. Even though *pediculus capitis* is very common in Africa from earlier times until now [12–15], there are only a few studies on knowledge, attitude, and practice regarding head lice infestations especially in sub-Saharan Africa [16]. Above all, majority of the previous studies focused on the knowledge, attitude, and practice of parents, nurses, and teachers only [17–21]. Let alone the level of knowledge about human head lice and their control in high-income countries is restricted in resource-constrained settings among schoolchildren [16, 19, 20, 22] and even insufficient among health professionals [23–25]. The major setbacks for ineffective control of *pediculus capitis* include lack of knowledge, undesirable attitude towards control and prevention of head lice, and inadequate personal hygiene practice [26–28].

Therefore, the present study was undertaken to assess the level of knowledge, attitude, practice, and their associated factors regarding *pediculus capitis* prevention and control among schoolchildren in Woreta town, northwest Ethiopia.

## 2. Methods

### 2.1. Study Design and Setting

This was a school-based cross-sectional study conducted from April to June 2018 among schoolchildren in Woreta town primary schools. The town is located 589 km far from Addis Ababa, the capital city of Ethiopia. During the study period, there were three primary schools in the town with a total of 3239 students. The prevalence of *pediculus capitis* during the study period was 65.7% (95% CI 60.01–70.3%) [29].

### 2.2. Sample Size Calculation and Sampling Procedure

For the present study, the sample size was determined using a single population proportion formula [30]. With the following assumptions: *p* = 50 percent to allow maximum variation (as there was no previous country analysis on the ratio of knowledge, attitude, and practice to *pediculus capitis* prevention and control), 95 percent confidence level, *z* = standard normal tabulated value, and *α* = level of significance and margin of error (*d*) = 0.05;

(1) $$\begin{array}{c} n=\frac{{\left(z_{\alpha /2}\right)}^{2}p\left(1-p\right)}{d^{2}}=\frac{{\left(1.96\right)}^{2}0.5\left(1-0.5\right)}{0.05^{2}}=384 \end{array}$$

The final total sample size was 402, after adding the estimated nonresponse rate of 5 percent. Study participants were chosen using a simple random sampling technique and distributed in proportion to the three schools, based on the number of students in each school.

### 2.3. Data Collection Tool and Quality Control Procedures

A pretested, semistructured questionnaire was used that included socio-demographic variables, knowledge, attitude, and practice items relevant to infestation with pediculus capitis. Two Environmental Health Bachelor's degree students conducted an interview and observation after receiving training on the data collection method, techniques, study intent, and ethical considerations.

Detailed explanation and reliability of the method for data collection based on the pretest results are discussed elsewhere [29].

### 2.4. Variable Measurement

#### 2.4.1. Pediculusis

A child with at least one head louse by wet combing is assumed to be infested with pediculus capitis [14].

#### 2.4.2. Schoolchildren

In the current report, children attending classes from grades 1 to 4 were considered schoolchildren [29].

#### 2.4.3. Knowledge

Knowledge was measured by 10 yes/no category knowledge items.

Students who scored mean of knowledge questions and above were deemed to have good knowledge [29].

#### 2.4.4. Attitude

Attitude was assessed by 8 Likert-scale attitude questions (1-strongly disagree with 5-strongly agree). Children who scored mean and above questions about the attitude were known as having a good attitude [29].

#### 2.4.5. Practice

Children were asked five specific questions regarding the prevention behavior of pediculus capitis. Those children who scored mean and above of the questions were considered good practice [29].

### 2.5. Data Analysis

Completeness and consistency of the data were reviewed regularly. Epi info version 7.1 was used for data entry and analysis was performed using SPSS version 21. We calculated the number, mean, and standard deviation to show the descriptive results.

Bivariable binary logistic regression was first tested, and in the final model, we used multivariable binary logistic regression to test variables with a *p* value <0.2 in bivariable analysis for significant association. Variables with a *p* value <0.05 were declared as being significantly associated with dependent variables (i.e., knowledge, attitude, and practice) in the multivariable binary logistic regression analysis.

Crude and adjusted odds ratios and 95% confidence intervals were calculated. Model fitness was tested using Hosmer and Lemeshow good-ness of fit at *p* > 0.05.

### 2.6. Patient and Public Participation

The current research did not include patients or the public but included students with and without*pediculus capitis* infestation.

## 3. Results

### 3.1. Socio-Demographic Characteristics

Four hundred and two students were included in the current study. Above half (53.7%) of the study participants were females. The study participants' mean age was 10.19 (±1.62) years. Almost all (93%) of the students attend in schools with no water access (Table 1).

**Figure 1:**
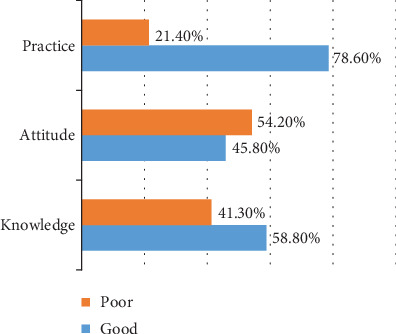
Knowledge, attitude, and practice towards *Pediculus capitis* prevention and control among schoolchildren Woreta, 2018 (*n* = 402).

From the total participants, 78.6% had good self-reported practice, 58.8% had good knowledge, and 45.8% had desirable attitude (Figure 1).

### 3.2. Correlation between Knowledge, Attitude, and Practice

The correlation between knowledge, attitude, and practice was assessed using the rank correlation coefficient of Spearman and represented using 0–0.25 = poor, 0.25–0.5 = average, 0.5–0.75 = good, and above 0.75 = excellent correlations based on criteria developed for the study of statistical power for behavioral sciences [31]. There was a significant correlation among knowledge, attitude and practice (Table 2).

### 3.3. Factors Associated with Knowledge regarding *pediculus capitis* Prevention

Age of child, paternal and maternal education, number of students per class, family size, water accessibility, attitude of students towards *pediculus capitis* prevention, practice towards *pediculus capitis* prevention, and previous history of *pediculus capitis* infestation were variables candidate for the multivariable binary logistic regression analysis as they have *p* value less than 0.2. Only age, attitude towards *pediculus capitis* prevention, practice towards *pediculus capitis* prevention, and *pediculus capitis* infestation were associated with knowledge regarding *pediculus capitis* prevention during the multivariable binary logistic regression analysis. Children aged 9 to 11 years had 2.24 times [AOR = 2.24, 95% C.I (1.10, 4.55)], and those aged ≥12 years had 3.84 times [AOR = 3.84, 95% C.I (1.56, 9.46)] better *pediculus capitis* prevention knowledge than those aged 5 to 8 years. Schoolchildren with good *pediculus capitis* prevention practice had 2.93 times better knowledge as compared to those with poor practice [AOR = 2.93, 95% C.I (1.39, 6.18)]. Study participants with previous history of *pediculus capitis* had twice better preventive knowledge than those with no history of infestation [AOR = 2.25, 95% C.I (1.14, 4.44)] (Table 3).

Students' knowledge towards *pediculus capitis* prevention, practice towards *pediculus capitis* prevention, and previous history of *pediculus capitis* infestation were factors associated with attitude about *pediculus capitis* prevention during the multivariable logistic regression (Table 4).

Grade level, paternal and maternal educational status, number of students per classroom, family size, knowledge and attitude towards *pediculus capitis* prevention, and history of infestation were factors eligible (*p* < 0.2) for multivariable analysis in the final model. The only number of students per classroom, family size, knowledge, attitude, and history of infestation was associated with *pediculus capitis* prevention practice during the multivariable analysis. Class size was associated with practice regarding *pediculus capitis* prevention. Students attending in larger size class had better prevention practice compared with those in smaller size classes. Students from lower family size had better *pediculus capitis* prevention practice [AOR: 2.37, 95% C.I (1.24, 4.54)]. Children with good knowledge had 2.93 times better practice than those with poor knowledge [AOR: 2.93, 95% C.I (1.32, 6.50)]. Study participants with desirable attitudes had 4.24 times better prevention practice than those with undesirable attitudes [AOR: 4.24, 95% C.I (1.60, 11.23)]. Children with previous history of infestation by *pediculus capitis* had better prevention practice [AOR: 3.52, 95% C.I (1.22, 10.15)] (Table 5).

## 4. Discussion

In this study, we evaluated the level of knowledge, attitude, and practice regarding *pediculus capitis* prevention and control among schoolchildren in Woreta town. This study is a part of a project on a school health assessment program, and detailed information about prevalence and risk factors of *pediculus capitis* is published elsewhere [29]. In the current manuscript, we have assessed the knowledge, attitude, and practice regarding *pediculus capitis* prevention and control and their associated factors. Knowledge was a significant predictor of infestation status in previous studies [19, 29, 32]. This may be due to the lack of knowledge that can result in insufficient capacity to handle lice infestation. Several interventional studies concentrated on health education intervention to improve knowledge of school teachers, guardians, and students [33, 34]. Deficiencies in knowledge may indicate inabilities to manage infestation. In the current analysis, the positive associations between knowledge-attitude, knowledge-practice, and attitude-practice notify the relationship among knowledge, attitude, and practice about *pediculus capitis* prevention. Sufficient knowledge will result in a positive attitude leading to better practice. This is in line with previous studies [35, 36]. Students with higher age, better attitude and practice, and those with no previous history of infestation had better-adjusted odds of knowledge regarding *pediculus capitis* prevention. Students with higher age were less likely to be infested [5]. In the current study, students with higher age were found to have better infestation prevention and control knowledge. This is in line with earlier studies [37].

Family size less than five, better knowledge and desirable attitude, and not being infested by *pediculus capitis* were among factors associated with practice regarding *pediculus capitis* prevention among schoolchildren. Previous research showed that children from lower socioeconomic classes and those with lower-educated parents were more frequently infested [38–43]. Greater family size was identified as a determinant for *pediculus capitis* in several earlier studies [44–46].

In the current study, students with better knowledge and desirable attitude more likely reported better *pediculus capitis* prevention and control practice. Health practice is defined by the knowledge and attitude of an individual or the public [37].

This research was, ultimately, not without limitations. No evaluation was made of the knowledge, attitude, and practice of school teachers and parents. The scarcity of previous studies on knowledge, attitude, practice, and associated factors among children made comparison of results difficult. An additional limitation of this study has been the inherent weakness of cross-sectional research design in determining cause-effect relationship, recall and social desirability bias, and poor generalizability as the analysis is performed only at a specific city.

## 5. Conclusion

Head lice infestation is a major public health concern and the national and regional health authorities need to advocate awareness-raising programs that target mothers and prepare knowledge, attitude, and practice improvement strategies.

## Figures and Tables

**Table 1:** Explanatory variables of school children Woreta town, 2018 (*n* = 402).

Variables	Categories	Frequency	Percent (%)
Student grade level	Grade1	99	24.6
	Grade2	99	24.6
	Grade3	100	24.9
	Grade4	104	25.9

Sex	Male	186	46.3
	Female	216	53.7

Age	5-8	85	21.1
	9-11	230	57.2
	>12	87	21.6

Religion	Orthodox	291	72.4
	Muslim	108	26.9
	Protestant	3	0.7

Fathers education	Illiterate	111	27.6
	Elementary	161	40
	Secondary and above	130	32.3

Mothers education	Illiterate	171	42.5
	Elementary	162	40.3
	Secondary and above	69	17.2

Fathers occupation	Private worker	153	38.1
	Government worker	64	15.9
	Daily labor	42	10.4
	Others	143	35.6

Mother's occupation	Private worker	62	15.4
	Government worker	25	6.2
	Housewife	240	59.7
	Others	75	18.7

Family size	≤5	227	56.4
	>5	175	45.6

Personal hygiene control	Yes	106	26.4
	No	296	73.6

Water access	Yes	28	7
	No	374	93

Average number of students per classroom	40-50	116	28.9
	51-56	121	30.1
	57-58	89	22.1
	59-67	76	18.9

**Table 2:** Correlation between knowledge, attitude, and practice towards *Pediculus capitis* prevention and control among schoolchildren Woreta, 2018 (*n* = 402).

Variables	Correlation coefficient	*p* value∗
Knowledge-attitude	0.629	<0.001
Knowledge-practice	0.363	<0.001
Attitude-practice	0.370	<0.001

∗Correlation significant at *p* < 0.01.

**Table 3:** Factors associated with knowledge regarding *pediculus capitis* prevention and control among schoolchildren Woreta, 2018 (*n* = 402).

Variables	Categories	Knowledge		COR (95% CI)	AOR (95% CI)
		Good (%)	Poor (%)		
Age in years	5 to 8	37 (43.5)	48 (56.5)	1	1
	9 to 11	136 (59.1)	94 (40.9)	1.88 (1.14, 3.10)	2.24 (1.10, 4.55)∗
	≥12	63 (72.4)	24 (27.6)	3.41 (1.80, 6.43)	3.84 (1.56, 9.46)∗∗

Fathers education	Illiterate	53 (47.7)	58 (52.3)	1	1
	Elementary	93 (57.8)	68 (42.2)	1.50 (0.92, 2.43)	1.18 (0.55, 2.56)
	Secondary and above	90 (69.2)	40 (30.8)	2.46 (1.45, 4.17)	1.73 (0.65, 4.64)

Mothers education	Illiterate	88 (51.5)	83 (48.5)	1	1
	Elementary	99 (61.1)	63 (38.9)	1.48 (0.96, 2.29)	0.80 (0.37, 1.72)
	Secondary and above	49 (71)	20 (29)	2.31 (1.27, 4.21)	0.73 (0.24, 2.22)

Number of students per class	40-50	78 (67.2)	38 (32.8)	2.03 (1.12, 3.67)	1.77 (0.76, 4.16)
	51-56	62 (51.2)	59 (48.8)	1.05 (0.59, 1.86)	0.68 (0.29, 1.61)
	57-58	58 (65.2)	31 (34.8)	1.87 (1.00, 3.50)	0.96 (0.38, 2.45)
	59-67	38 (50)	38 (50)	1	1

Family size	≤5	146 (64.3)	81 (35.7)	1.70 (1.14, 2.55)	1.00 (0.56, 1.80)
	>5	90 (51.4)	85 (48.6)	1	1

Water accessibility	Yes	13 (46.4)	15 (53.6)	0.59(0.27, 1.27)	0.75 (0.26, 2.16)
	No	223 (59.6)	151 (40.4)	1	1

Attitude towards pediculus capitis prevention	Poor	66 (30.3)	152 (69.7)	1	1
	Good	170 (92.4)	14 (7.6)	27.96(15.09, 51.82)	16.95 (8.74, 32.88)∗∗∗

Practice towards *pediculus capitis* prevention	Poor	21 (24.4)	65 (75.6)	1	1
	Good	215 (68)	101 (32)	6.59 (3.82, 11.37)	2.93 (1.39, 6.18)∗∗

*Pediculus capitis* infestation	Yes	116 (84.1)	22 (15.9)	1	1
	No	120 (45.5)	144 (54.5)	6.33 (3.78, 10.6)	2.25 (1.14, 4.44)∗

Significant at ∗*p* ≤ 0.05, ∗∗*p* ≤ 0.01, ∗∗∗*p* ≤ 0.001, Hosmer-Lemeshow goodness-of-fit (*p* = 0.196) 1 reference.

**Table 4:** Factors associated with attitude regarding *pediculus capitis* prevention among schoolchildren Woreta, 2018 (*n* = 402).

Variables	Categories	Attitude		COR (95% CI)	AOR (95% CI)
		Good (%)	Poor		
Age in years	5 to 8	30 (35.3)	55 (64.7)	1	1
	9 to 11	105 (45.7)	125 (54.3)	1.54 (0.92, 2.58)	1.18 (0.56, 2.46)
	≥12	49 (56.3)	38 (43.7)	2.36 (1.28, 4.37)	1.47 (0.61, 3.57)

Fathers education	Illiterate	35 (31.5)	76 (68.5)	1	1
	Elementary	76 (47.2)	85 (52.8)	1.94 (1.17, 3.22)	1.87 (0.84, 4.17)
	≥secondary	73 (56.2)	57 (43.8)	2.78 (1.64, 4.71)	1.65 (0.61, 4.44)

Mothers education	Illiterate	64 (37.4)	107 (62.6)	1	1
	Elementary	78 (48.1)	84 (51.9)	1.55 (1.00, 2.40)	0.72 (0.34, 1.53)
	≥secondary	42 (60.9)	27 (39.1)	2.6 (1.46, 4.62)	0.90 (0.38, 2.20)

Number of students per class	40-50	56 (48.3)	60 (51.7)	1.49 (0.82, 2.68)	0.78 (0.34, 1.81)
	51-56	51 (42.1)	70 (57.9)	1.18 (0.66, 2.12)	0.92 (0.38, 2.20)
	57-58	48 (53.9)	41 (46.1)	1.90 (1.02, 3.54)	0.96 (0.39, 2.40)
	59-67	29 (38.2)	47 (61.8)	1	1

Family size	≤5	119 (52.4)	108 (47.6)	1.86 (1.25, 2.79)	1.19 (0.67, 2.12)
	>5	65 (37.1)	110 (62.9)	1	1

Knowledge towards *pediculus capitis* prevention	Poor	14 (8.4)	152 (91.6)	1	1
	Good	170 (72)	66 (28)	27.96 (15.09, 51.82)	17.32 (8.90, 33.72)∗∗∗

Practice towards *pediculus capitis* prevention	Poor	9 (10.5)	77 (89.5)	1	1
	Good	175 (55.4)	141 (44.6)	10.62 (5.14, 21.93)	4.33 (1.69, 11.09)∗∗

*pediculus capitis* infestation	Yes	81 (30.7)	183 (69.3)	1	1
	No	103 (74.6)	35 (25.4)	6.65 (4.18, 10.58)	2.97 (1.64, 5.36)∗∗∗

Significant at ∗*p* ≤ 0.05, ∗∗*p* ≤ 0.01, ∗∗∗*p* ≤ 0.001, Hosmer-Lemeshow goodness-of-fit (*p* = 0.134). COR: crude odds ratio; AOR: adjusted odds ratio; CI: confidence interval.

**Table 5:** Factors associated with *pediculus capitis* prevention practice among schoolchildren Woreta, 2018 (*n* = 402).

Variables	Categories	Practice		COR (95% CI)	AOR (95% CI)
		Good (%)	Poor (%)		
Grade level	Grade 1	76 (76.8)	23 (23.2)	1	1
	Grade 2	66 (66.7)	33 (33.3)	0.61 (0.32, 1.13)	0.32 (0.13, 0.76)
	Grade 3	79 (79)	21(21)	1.14 (0.58, 2.23)	0.62 (0.25, 1.52)
	Grade 4	95 (91.3)	9 (8.7)	3.19 (1.40, 7.31)	0.57 (0.18, 1.80)

Fathers education	Illiterate	72 (64.9)	39 (35.1)	1	**1**
	Elementary	125 (77.6)	36 (22.4)	1.88 (1.10, 3.22)	0.92 (0.43, 1.98)
	Secondary and above	119 (91.5)	11 (8.5)	5.86 (2.82, 12.16)	1.46 (0.47, 4.50)

Mothers education	Illiterate	113 (66.1)	58 (33.9)	1	1
	Elementary	137 (84.6)	25 (15.4)	2.81 (1.65, 4.78)	1.29 (0.56, 2.92)
	≥secondary	66 (95.7)	3 (4.3)	11.29 (3.40, 37.47)	5.36 (0.98, 29.36)

Number of students per class	40-50	92 (79.3)	24 (20.7)	2.94 (1.55, 5.57)	2.20 (0.90, 5.38)
	51-56	99 (81.8)	22 (18.2)	3.45 (1.81, 6.60)	4.61 (1.83, 11.67)∗∗∗
	57-58	82 (92.1)	7 (7.9)	8.99 (3.67, 22.01)	8.18 (2.73, 24.46)∗∗∗
	59-67	43 (36.6)	33 (43.4)	1	1

Family size	≤5	197 (86.8)	30 (13.2)	3.09 (1.88, 5.09)	2.37 (1.24, 4.54)∗∗
	>5	119 (68)	56 (32)	1	1

Knowledge towards *pediculus capitis* prevention	Poor	101 (60.8)	65 (39.2)	1	**1**
	Good	215 (91.1)	21 (8.9)	6.56 (3.82, 11.37)	2.93 (1.32, 6.50)∗∗

Attitude towards *pediculus capitis* prevention	Poor	141 (64.7)	77 (35.3)	1	1
	Good	175 (95.1)	9 (4.9)	10.62 (5.14,21.93)	4.24 (1.60, 11.23)∗∗

*Pediculus capitis* infestation	Yes	184 (69.7)	80 (30.3)	1	1
	No	132 (95.7)	6 (4.3)	9.56 (4.05, 22.57)	3.52 (1.22, 10.15)∗

Significant at ∗*p* ≤ 0.05, ∗∗*p* ≤ 0.01, ∗∗∗*p* ≤ 0.001, Hosmer-Lemeshow goodness-of-fit (*p* = 0.478) 1 reference.

## Data Availability

The datasets available from the corresponding authors upon reasonable request.

## Abbreviations

AOR: Adjusted odds ratio
COR: Crude odds ratio
CI: Confidence interval
EPI Info: Epidemiological information
SPSS: Statistical Package for Social Sciences.
